# Chicago Medical Society

**Published:** 1888-06

**Authors:** 


### CHICAGO MEDICAL SOCIETY.

STATED MEETING, JANUARY 3, 1888.

The President, W. T. Belfield, in the chair.

Dr. Henry Gradle read a paper on "Morbid Nasal Irritability," which, he said, is a condition of morbid sensibility of the nasal surfaces associated with hypertrophy of the submucous cavernous tissue. The condition is confounded in text-books with chronic catarrh. The best account of it is in Mackenzie's article on neuroses of the nose, in the "Reference Handbook of Medical Sciences." The local reflexes form the basis of the present paper.

There is, first, the vascular reflex. The vascular or cavernous tissue normally developed to a slight extent at the front and rear end of the inferior turbinated bone, is enlarged and augmented, and may appear also on the side of the septum, on the floor of the nose, and on the front end of the middle turbinated bone. Through any irritation by dust or chilling of the surface this vascular plexus becomes engorged, and if well developed in proportion to the size of the nasal cavity it obstructs the passage. It is the temporary or fleeting character of the obstruction which characterizes the irritable nose, not any permanent narrowing of the passage. Almost invariably engorgement occurs on one side at a time, and then may jump over to the other.

Dependent upon this vascular dilatation is the second reflex, namely, sneezing. With the sneezing there occurs a third reflex, namely, copious secretion of clear mucus, indicating the absence of any inflammatory condition. This irritable nose may lead to reflexes in distant regions, as to the eyes, ears, throat or nervous system at large. The irritable nose may be a symptom of some other nasal disease, such as catarrh or the presence of polypi ; it can also be caused by large irritated tonsils. But in many cases it exists by itself without any other primary disease to which it could be traced. It is very much influenced by the climate, and patients can get relief by going to the mountains of the West or to the Pacific coast.

In the treatment any traceable causes, such as tangible diseases of the tonsils, should first be removed. Quinine relieves the attacks, but probably does not cure them. No other drugs have seemed to him to be of any service. Where the condition is not symptomatic of another disease of the nose the only cure is the destruction of the cavernous tissue by the galvano-cautery, or in milder cases, by chromic acid. The latter agent is not as reliable as the former. A cure is possible whenever the extent of hypertrophied cavernous tissue is circumscribed, while where it is very diffuse, relief, but not an absolute cure, can be promised.

Professor N. S. Davis, Jr., reported a case of " Rupture of an Aortic Valve. "

Four months previous to entering the hospital the patient was carrying a heavy load of coal up stairs when suddenly he felt something give way in his left side ; he experienced considerable dyspnœa at once, and symptoms of cardiac insuffiency date from that time. At first the dyspnœa was noticeable chiefly on walking or on making rapid movements, but it was sufficient to necessitate his giving up all work. The dyspnœa increased steadily, and he became very markedly anæmic, and in addition exhibited signs of general œdema. At the time he entered the hospital there was ascites as well as extensive anasarca of the extremities. He was unable to lie with any degree of comfort, but had constantly to sit. The pulse was exceedingly feeble and very rapid.

A collection of fluid was found in one of the pleural cavities. The lungs were evidently œdematous. The action of the heart was rapid and feeble ; the cardiac sounds were distant. There was present over both the mitral and aortic areas a regurgitant murmur. The area of cardiac dullness was greatly increased in all directions. There was no prior history of rheumatic trouble. The liver was considerably enlarged. A little later, on the day of admission, the house physician noticed that the patient was sleeping almost constantly, and that the heart's action was very much more feeble, and that the respiratory motions were only eleven to the minute. On arousing the patient the respiratory movements improved somewhat. On the following morning the patient was more drowsy, stupid, and sleepy. In the afternoon of that day he exclaimed suddenly that he felt faint, and with a gasp or two was dead.

The post-mortem revealed a small amount of liquid in one pleural cavity. The lungs were œdematous and quite firm in the lower and posterior parts from chronic venous hyperæmia. The liver was enlarged, unusually hard, and full of blood; the spleen normal; the left kidney normal; the right kidney had a slightly adherent capsule. The heart was greatly enlarged. In the pericardial sac there was an ounce and a half of fluid. Both of the ventricles were dilated without hypertrophy. On the right side the valves are normal, on the left side the mitral valves are normal, but the aortic valves are thickened. Nowhere is ulceration or destruction of tissue from that cause to be seen. The valves are not roughened. The part of the valve where there is loss of continuity is also slightly thickened. The break in continuity here seen occurs in the edge of the valve just where it should be attached to the aorta. If rupture took place, necessarily it took place four months before the death of the patient, and, naturally, thickening of the torn edges might occur. There is also present in the aorta above the valves small areas of thickening and hardening. Taking into consideration both the morbid appearances and the history of the case, it is most probable that four months before death, at the time from which the patient uniformly dated his trouble, a rupture of the aortic valve occurred.

Dr. Norval H. Pierce reported a case of "Operation for Hæmothorax."

The patient, male, 19 years old, previously in good health, accidentally stabbed himself in the third intercostal space, half an inch to the left of the parasternal line. The wound was made with a knife-blade two inches long and half an inch wide. Dr. Pierce first saw the patient three and a half hours after the accident. He had had no treatment, and his condition is described as follows: Pale features, pinched, eyes sunken, and pupils widely dilated, surface cold and clammy, impaired hearing, pulse nearly imperceptible and very rapid, respiration sighing. There was very slight external bleeding at the wound, which was about an inch in length. From an inch above the sternum to the lower border of the liver the right side was dull, but at this time the liver was not displaced. No blood was expectorated. The wound, was closed antiseptically, an ice-bag ordered for the chest, warm bottles to the lower extremities, head lowered, whisky, half ounce every forty-five minutes, together with ergot, very little liquids, and absolute rest.

The pulse improved gradually, but the anæmia persisted, dyspnœa arose, together with great restlessness and troublesome insomnia, and the right pleural cavity remained flat on percussion; the lower border of the liver gradually descended, and the heart was displaced to the left. At this time the advisability of operative interference was discussed, but it was not undertaken.

On July 27 a gallon of dark-colored blood was drawn away. This did not cause the dullness over the right thoracic area to disappear, but relieved the difficult breathing and lessened the restlessness and insomnia.

On August 20, the lower border of the liver being within an inch of the ilac crest, another gallon was drawn away. Up to this time the temperature had ranged from 100° to 101° Fahr. Afterwards it fell to normal once or twice, but generally the thermometer registered 100° Fahr.

On September 5 diarrhœa set in, and lasted two or three days. Cough developed soon after, with very offensive expectoration. The line of the liver dullness was gradually descending, and extreme dyspnœa began. On September 23 there was a marked lowering of his general condition, and the operation for empyæma was attempted, but the patient bore the ether so badly that it was abandoned. The tissues of the seventh intercostal were therefore simply incised, about a gallon of fluid allowed to escape, and the opening plugged with iodoform gauze. At the end the patient was in a state of collapse. He rallied, however, and the next day a tube was inserted and the cavity washed with a saturated solution of boracic acid at 100° Fahr., which was repeated every second day thereafter. Fever and cough disappeared on the second day. The washings grew less and less colored, and in a few days the tube was removed.

As regards the place for the incision, the seventh intercostal space is too low, as after the first week the movements of the diaphragm interfered with the drainage, tube.

DISCUSSION.

Professor A. E. Hoadley thought that the freedom of the hæmorrhage and absence of hæmoptysis indicated a wound of the internal mammary artery. He thought the hæmorrhage could have been controlled by introducing a button into the wound, such a one as would go through the wound with a string attached to it, pushing it into the thoracic cavity, and then by making traction on the string outside, it would have closed the bleeding vessels. As far as the tolerance of the pleural cavity of blood is concerned, there is no difference between the two cavities—abdominal and thoracic.

Professor W. T. BELFIELD exhibited a specimen of "Muscle Infiltrated with Trichinæ." It was a piece of gastrocnemius muscle from the leg of a man which was amputated because of disease of the knee joint. Some eighteen years ago this man, in company with a number of other individuals, ate of some poorly cooked ham and sausage; nearly all of those who partook of this article of diet became very ill within a week, and a large percentage died. This man was very ill for two months, and did not entirely recover his health for some four months. This man recovered, and during the succeeding 18 years has enjoyed good health, except that he has at various times, and more particularly during the last six or eight years, suffered from rheumatism in different parts of the body.

This muscle, like all the other muscles of the amputated leg, is thickly studded with trichinæ.

